# Computed tomography-guided preoperative localization of musculoskeletal lesions using the ROLL technique

**DOI:** 10.1590/0100-3984.2017.0010

**Published:** 2018

**Authors:** Chiang Jeng Tyng, Paula Nicole Vieira Pinto Barbosa, Almir Galvão Vieira Bitencourt, Maurício Kauark Amoedo, Maria Fernanda Arruda Almeida, Eduardo Nóbrega Pereira Lima, Rubens Chojniak

**Affiliations:** 1 MD, PhD, Department of Imaging, A.C.Camargo Cancer Center, São Paulo, SP, Brazil.; 2 MD, MSc, Department of Imaging, A.C.Camargo Cancer Center, São Paulo, SP, Brazil.

**Keywords:** Nuclear medicine, Gamma cameras, Medical oncology, Musculoskeletal system, Minimally invasive surgical procedures

## Abstract

**Objective:**

To describe the preoperative localization of musculoskeletal lesions with the radioguided occult lesion localization (ROLL) technique.

**Materials and Methods:**

In all cases, computed tomography-guided injection of technetium-99m sulfur colloid was performed, directly into or near the suspicious lesion, up to 36 hours before the surgical procedure. Lesions were detected intraoperatively with a gamma probe.

**Results:**

We report the cases of six patients submitted to radioguided surgery, including three patients with bone lesions suspicious for metastasis, two patients suspected of recurrent sarcoma, and one patient with no previous diagnosis who had a nodular lesion on the left leg. Patients tolerated the procedure well, and no complications were associated with the puncture. All marked lesions were easily identified intraoperatively and were excised with clear margins.

**Conclusion:**

The ROLL technique was effective in the intraoperative localization of occult musculoskeletal lesions, demonstrating that it is a feasible and promising technique for the surgical exploration of selected cases.

## INTRODUCTION

Radioguided surgery has proven to be a safe and effective alternative for the management of cancer patients with small nonpalpable lesions^(^^1^^)^. The radioguided occult lesion localization (ROLL) technique can be useful in selected cases when suspicious lesions may be difficult to identify intraoperatively due to their dimensions or anatomical location. In such cases, wide excision and extensive exploration are required. However, those procedures can be unnecessarily traumatic and time-consuming. Preoperative localization allows more conservative excision and targeted resection, reducing surgery-related morbidity. The ROLL technique under imaging guidance has routinely been used at many specialized centers for the preoperative localization of occult breast lesions. However, few studies have reported the use of this technique for other pathologies^(^^2^^,^^3^^)^.

The aim of this study was to describe the preoperative localization of musculoskeletal lesions using the ROLL technique in patients undergoing radioguided surgery.

## MATERIALS AND METHODS

Lesions were localized by means of computed tomography (CT)-guided administration of radiotracer in real time and through direct puncture of the lesion, under local anesthesia. Injection of 0.1-0.2 mL of technetium-99m sulfur colloid with an activity count of 0.5-1.0 mCi (18.5-37.0 MBq) was administered directly into or next to the suspect lesion up to 36 hours prior to surgery. Immediately after injection of the radioactive material, single photon emission computed tomography/CT (SPECT/CT) imaging was performed to confirm the location and technical quality of the injection. Lesions were detected intraoperatively with a gamma probe. The radioactivity of the tissue was measured *in vivo* and after excision, as was the radioactivity of the surgical bed, to confirm that the lesion marked had been fully excised.

## RESULTS

**Figure 1:**
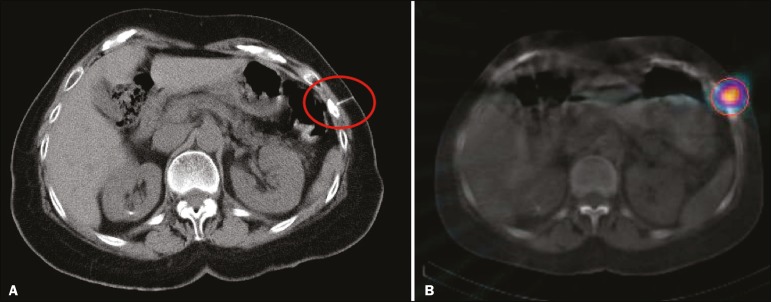
A 62-year-old woman with rectal adenocarcinoma, lung metastasis, and a nonspecific textural change in a left rib. **A:** Preoperative CT-guided injection of technetium-99m sulfur colloid adjacent to the lesion on the left rib. **B:** SPECT/CT confirming the location of the injection.

**Figure 2:**
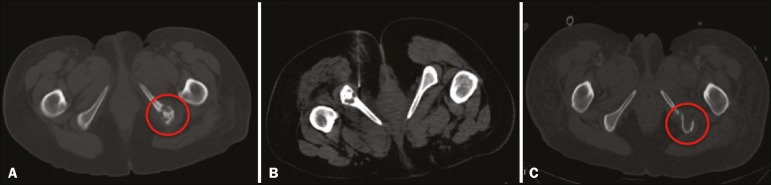
A 54-year-old woman with endometrial carcinoma. **A:** Non-contrast-enhanced CT with the patient in the supine position showing a lytic lesion on the left ischium. **B:** Preoperative CT-guided injection of technetium-99m sulfur colloid adjacent to the bone lesion with the patient in the prone position. **C:** Follow-up CT with the patient in the supine position after resection of the lesion on the left ischium.

**Figure 3:**
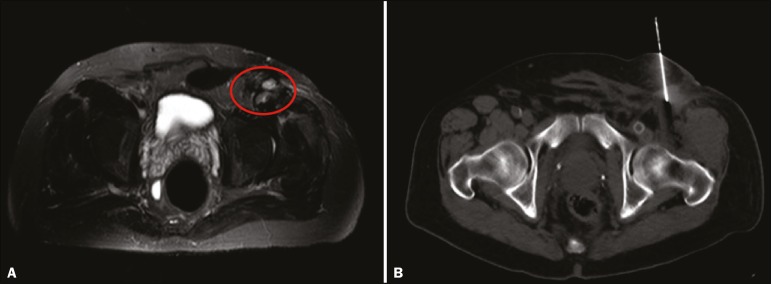
A 63-year-old man with history of inguinal liposarcoma and multiple local recurrences. **A:** Magnetic resonance imaging showing suspicious nodules at the surgical site. **B:** Preoperative CT-guided injection of technetium-99m sulfur colloid near the nodules.

We report the cases of six patients: three with bone lesions suspicious for metastasis (Figures 1 and 2); two with suspected recurrent sarcoma (Figure 3); and one with no previous diagnosis who had a nodular lesion on the left leg. Table 1 outlines the clinical histories, lesion locations, and pathological findings after surgical resection.

**Table 1:** Clinical data, lesion characteristics, and pathological results for all lesions submitted to surgical resection with the ROLL technique.

Patient	Primary tumor	Lesion type and localization	Pathological finding
44-year-old male	Retroperitoneal liposarcoma	Follow-up CT showed a lytic lesion on the right seventh rib, with increased uptake on bone scintigraphy	Fibrous dysplasia
63-year-old male	Inguinal liposarcoma	Magnetic resonance imaging and PET/CT showed a suspicious nodules at the surgical site	Recurrent sarcoma
54-year-old female	Endometrial adenocarcinoma	CT showed a lytic lesion on the left ischium	Metastatic, poorly differentiated adenocarcinoma
40-year-old female	No previous tumor	Magnetic resonance imaging showed an intramuscular lipomatous lesion of the left leg, with pain and edema	Intramuscular lipoma
84-year-old male	Right-thigh soft-tissue sarcoma	Magnetic resonance imaging showed a lesion suspected to be local recurrence	Spindle-cell sarcoma
62-year-old female	Rectal cancer with lung metastasis	CT showed nonspecific textural changes in the left ribs, with increased uptake on scintigraphy	Negative for metastatic disease

The procedure was well tolerated by the patients, with no complications associated with the puncture. The SPECT/CT images showed that the radiotracer was in the proper location. All marked lesions were easily identified intraoperatively and were excised with clear margins.

## DISCUSSION

Most bone and soft tissue lesions can be safely accessed through percutaneous imaging-guided biopsies^(^^4^^-^^8^^)^. In some cases, the percutaneous approach can be difficult, especially when the lesion is small or is located near blood vessels and nerves. In such cases, open surgical biopsy should be considered. However, intraoperative localization of the lesion may be time-consuming and unnecessarily traumatic, requiring extensive exploration^(^^9^^)^. Preoperative localization of the lesion could be the best option in these cases.

The ROLL technique is an extremely useful means of guiding surgical interventions for nonpalpable lesions and lesions located close to scar tissue or associated with distorted anatomy. In such situations, radioguided surgery facilitates targeted resection, decreasing operative time and morbidity. This procedure also increases the success rate of complete excision, reducing the number for reoperations^(^^2^^,^^3^^)^.

Several other techniques have been utilized to facilitate the localization of musculoskeletal lesions for biopsy. For bone lesions with increased uptake on scintigraphy, a gamma probe can be used in order to localize lesions intraoperatively after intravenous administration of technetium-99m-methylene diphosphonate in the preoperative period^(^^1^^)^. This method reduces the morbidity associated with the procedure. However, this technique depends on a brief operative time and is not useful for lesions without increased uptake on bone scan.

For lesions identified on positron emission tomography/CT (PET/CT), radioguided surgery using a PET-dedicated probe has been described. However, those probes are not as widely available as are gamma probes, the PET-probe technique is more complex because each tissue has different background uptake, and the technique exposes the surgical team to higher levels of radiation. García et al.^(^^10^^)^ compared the use of the PET-probe and ROLL techniques for intraoperative localization of lesions previously detected by PET/CT. The authors suggested that ROLL is the technique of choice for solitary lesions of easy percutaneous access with a biopsy needle and that a PET probe should be used only for lesions that are not eligible for the percutaneous approach.

Imaging-guided preoperative hook-wire localization is also routinely used for nonpalpable breast lesions and has been described for extramammary lesions. Brown et al.^(^^11^^)^ described 16 procedures using this method and showed that it can minimize the operative time and reduce the likelihood of reoperation. Morrison et al.^(^^9^^)^ used the same technique in five patients with musculoskeletal lesions, including three sclerotic rib lesions, one paraspinal soft-tissue lesion, and one popliteal soft-tissue lesion. Surgical excision was successful in all five cases.

For nonpalpable breast lesions, the ROLL technique has shown to be as effective as is the standard wire technique^(^^12^^)^. For the patient, ROLL is much more comfortable, causing less discomfort and pain; it is also faster and simpler to perform than is wire localization and produces lower positive margin rates and fewer reoperations^(^^12^^,^^13^^)^.

In conclusion, the ROLL technique was found to be effective in the intraoperative localization of nonpalpable musculoskeletal lesions and allowed accurate surgical excision. It is a feasible and promising technique for the surgical exploration of selected cases.
